# Magnetic resonance lymphangiography: Establishing normal

**DOI:** 10.1016/j.jvsv.2024.101870

**Published:** 2024-03-20

**Authors:** Mike Mills, Greta Brezgyte, Bernard Ho, Julian Pearce, Kristiana Gordon, Peter S. Mortimer, Pia Ostergaard, Franklyn A. Howe

**Affiliations:** a Neurosciencs and Cell Biology Research Institute, St. George's University of London, London, UK; b Cardiovascular and Genomics Research Institute, St. George's University of London, London, UK; c Dermatology and Lymphovascular Medicine, St. George's University Hospitals NHS Foundation Trust, London, UK

**Keywords:** Lower extremity, Lymphatic vessels, Lymphography, Magnetic resonance imaging

## Abstract

**Background:**

Despite an increased interest in visualizing the lymphatic vessels with magnetic resonance lymphangiography (MRL), little literature is available describing their appearance in nonlymphedematous individuals. To determine lymphatic abnormalities, an understanding of how healthy lymphatic vessels appear and behave needs to be established. Therefore, in this study, MRL of individuals without a history of lymphatic disease was performed.

**Methods:**

A total of 25 individuals (15 women) underwent MRL of their lower limbs using a 3.0 T Philips magnetic resonance imaging scanner (Philips Medical Systems). The first nine participants were recruited to establish the concentration of gadolinium-based contrast agent (GBCA) to administer, with the remainder imaged before and after interdigital forefoot GBCA injections at the optimized dose. Outcomes, including lymphatic vessel diameter, tortuosity, and frequency of drainage via particular drainage routes, were recorded.

**Results:**

Healthy lymphatic vessels following the anteromedial pathway were routinely observed in post-contrast T1-weighted images (average tortuosity, 1.09 ± 0.03), with an average of 2.16 ± 0.93 lymphatic vessels with a diameter of 2.47 ± 0.50 mm crossing the anterior ankle. In six limbs, vessels following the anterolateral pathways were observed. No vessels traversing the posterior of the legs were seen. In a subset of 10 vessels, the lymphatic signal, measured at the ankle, peaked 29 minutes, 50 seconds ± 9 minutes, 29 seconds after GBCA administration. No lymphatic vessels were observed in T2-weighted images.

**Conclusions:**

Contrast-enhanced MRL reliably depicts the lymphatic vessels in the legs of healthy controls. Following interdigital contrast injection, anteromedial drainage appears dominant. Quantitative measures related to lymphatic vessel size, tortuosity, and drainage rate are readily obtainable and could be beneficial for detecting even subtle lymphatic impairment.

**Article Highlights**

• **Type of Research:** A single-center, prospective, observational study
• **Key Findings:** Lymphatic vessels following the anteromedial and anterolateral pathways were observed in most healthy limbs imaged (30 of 31). The vessels were superficial and linear (average tortuosity, 1.09 ± 0.03 in the 27 vessels interrogated). Drainage of the contrast agent was slow, with signal often rising throughout the course of imaging.
• **Take Home Message:** The depiction of normal lymphatic vessels in nonlymphedematous limbs can be observed following gadolinium-based contrast injections in the forefoot, and metrics related to lymph drainage rate, lymphatic vessel size and tortuosity are measurable.

The lymphatic system is arguably the most neglected bodily system; thus, its contribution to human health and disease is poorly understood. Lymphedema, the chronic swelling of tissues due to a failure of the lymphatic system, is estimated to affect 140 to 250 million people worldwide but remains insufficiently characterized.^1^ Genes and molecular proteins specific to the lymphatic system have been discovered only relatively recently. This has enabled a greater understanding of lymphatic development and the active role of lymphatic vessels in cellular and physiological processes; however, knowledge of human lymphatic disease remains limited by a lack of reliable investigatory techniques. Blood vessels such as veins are visible to the naked eye and can be studied using noninvasive duplex ultrasound examinations. Lymphatic vessels are not easily seen and cannot be reliably imaged without the aid of an exogenous contrast medium.

Hudack and McMaster^2^ injected their skin with a blue dye in 1933, observing how quickly the dye flowed through their dermal lymphatic vessels, sparking an interest in imaging the lymphatic vessels. Following this, x-ray imaging after administration of a suitable contrast agent to cannulated lymphatic vessels facilitated visualization of larger collecting vessels. The first human lymphangiograms were produced by Kinmonth^3^ in 1952 and demonstrated lymphatic vessels from foot to groin. This resulted in a better understanding of many forms of lymphedema, especially primary lymphedema. Although direct contrast x-ray lymphography gives highly detailed images of collecting lymphatic vessels in the lower limbs, the procedure is invasive and not without risk (eg, lipiodol-induced embolism of the vessel) and now rarely used.^4^ Indocyanine green lymphography (ICG-L) provides in vivo real-time imaging of lymphatic vessels but is limited to imaging only the most superficial vessels. Lymphoscintigraphy (LS) is the current clinical investigation for diagnosis but functional, rather than anatomical, detail is provided. Magnetic resonance lymphangiography (MRL) can provide detail of the lymphatic vessels in greater detail than LS, without the associated ionizing radiation and is not limited by penetrance, a drawback of ICG-L.^5^

To determine what is abnormal, one must know what is normal. Despite the improved imaging detail, MRL has not facilitated the identification of lymphatic structures universally in those studies imaging nonaffected limbs; thus, what constitutes normal MRL findings is still unclear.^6^ In this report, we share our experience with identifying the lymphatic vessels with MRL in healthy subjects with normal lower limbs. Because this study is of healthy individuals, it provides a normal baseline for MRL, unlike studies of the contralateral limb of patients with unilateral lymphedema, a potentially problematic comparison group given that lymphatic dysfunction in these clinically asymptomatic limbs has been reported.^7^^,^^8^ Our aim was therefore to determine if MRL is a robust and tolerable method of visualizing lymphatic vessels and lymph nodes and able to establish their anatomy and function in individuals unaffected by lymphedema.

## Methods

### Participants

The healthy individuals included in this study were recruited as part of an ongoing program of research to improve understanding of the causal mechanisms and implications of primary lymphedema. This program includes investigations of the limb lymphatic vessels of patients with primary lymphedema and healthy individuals using MRL (approved by the London – Camden & Kings Cross research ethics committee; approval no., 20/LO/0237), with healthy participants (predominantly staff, students, and unaffected spouses of lymphedema patients) recruited and imaged to both improve our understanding of the anatomy and physiology of healthy limb lymphatic vessels and provide an approximately age- and sex-matched comparison group for the lymphedema cohort. This report focuses on improving the understanding of healthy lower limb lymphatic vessels and, thus, exclusively reports on participants without a lymphedema diagnosis. The participant demographics are summarized in Table I.

**Table I:** Study participant characteristics

Characteristic	Value
Age, mean ± SD years (range)	37.6 ± 11.2 (23.5-69.0)
Sex, *n* (%)	
Male	10 (40.0)
Female	15 (60.0)
Limbs excluded, No.	1

*SD,* Standard deviation.

### Injection protocol

All participants received a diluted mixture of 0.5 M gadolinium-based contrast agent (GBCA; Dotarem; Guerbet), saline, and 1% lidocaine anesthetic in 1-mL volumes per injection. Injections were performed intradermally in each of the four interdigital spaces of the foot, as is routine in the MRL literature,^6^^,^^9^ manually and at a slow rate (each injection typically lasting 15-60 seconds) by dermatologists.

### Dose optimization

**Fig 1:**
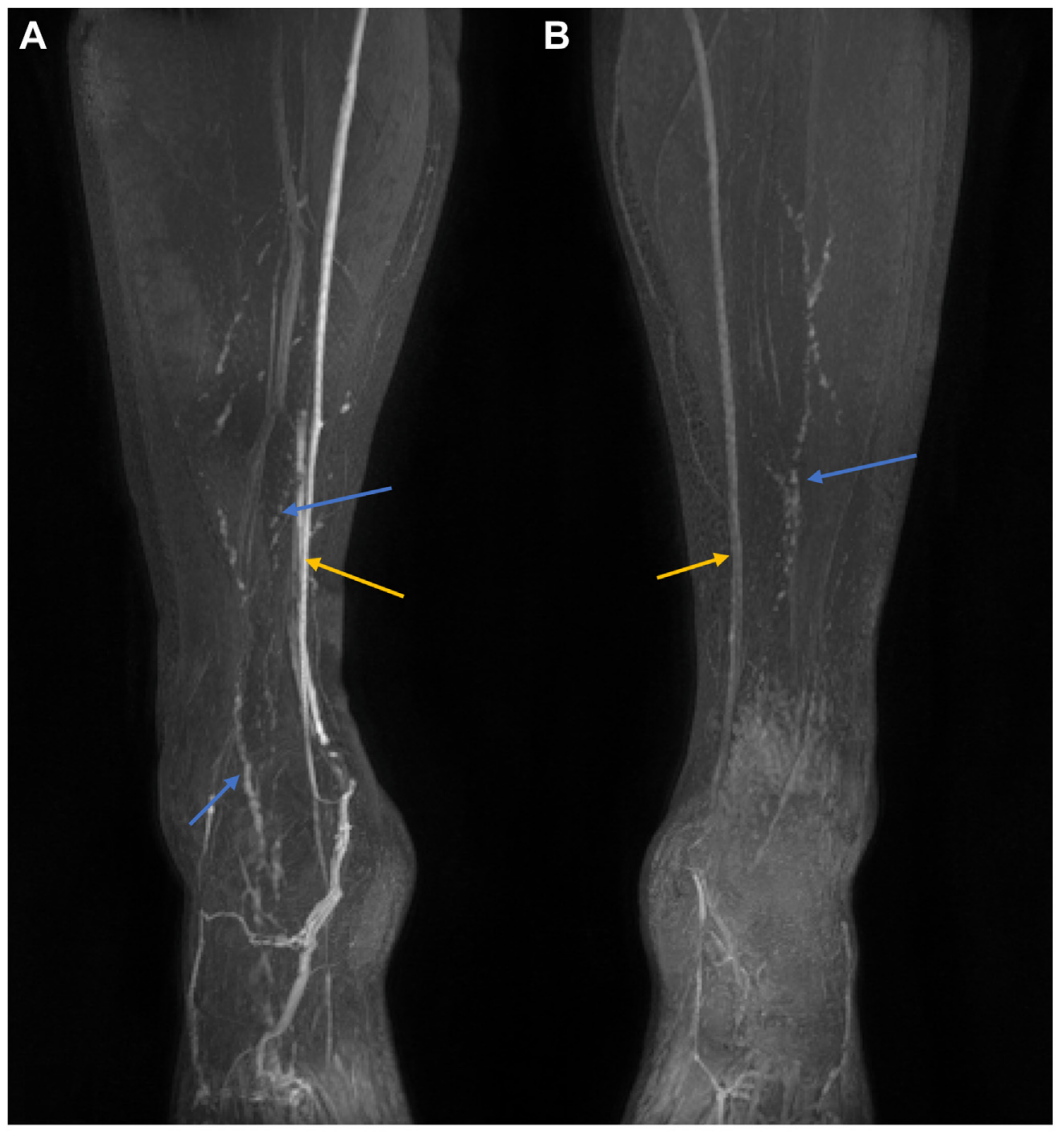
Injection protocol optimization detects differences in lymphatic vessel visibility. Bilateral coronal maximum intensity projection (MIP) images acquired in a 23-year-old woman. Four 1 mL interdigital contrast injections were administered into the dermis of each foot. The right leg (**A**) was injected with a gadolinium-based contrast agent (GBCA) at a concentration of 0.45 mL GBCA/mL and the left (**B**) with 0.1 mL GBCA/mL. In the limb receiving a higher GBCA concentration, both veins (*orange arrows*) and lymphatic vessels (*blue arrows*) appear brighter. Images are displayed with the same window and level and at approximately the same timepoint after contrast injection.

Nine participants (three men and six women) were initially enrolled to investigate the effect of the concentration of GBCA (either 0.02 mL/mL, 0.1 mL/mL, or 0.45 mL/mL of the volume administered) on the visibility of lymphatic vessels, with the concentration considered to deliver the clearest and most consistent lymphatic enhancement used thereafter. For some participants, a different GBCA concentration was administered to each limb to facilitate a direct contralateral comparison (Fig 1). This optimization was performed dynamically, such that if a GBCA dose was unable to depict the lymphatic vessels after multiple attempts, it was removed from consideration. Contrast injection concentrations are summarized in Table II.

**Table II:** Contrast injection details

Injection protocol optimisation	
Contrast volume / injection (mL)	# of limbs analysed (F/M)
0.45	7/3
0.1	4/2
0.02	1/1
Main study	
Contrast volume / injection (mL)	# of limbs analysed (F/M)
0.45	18/13

*F*, Female; *M*, male.

### Imaging protocol

All participants were imaged feet-first and supine using a 3.0 T system (Dual TX Achieva; Philips Medical Systems) with a 16-element torso receiver coil positioned around the leg from the ankle to the knee. The lower limbs were imaged before contrast administration with a heavily T2-weighted three-dimensional (3D) turbo-spin echo sequence and before and after contrast with moderately T1-weighted 3D spoilt gradient echo imaging (Table III). The T1-weighted sequence was repeated in a dynamic manner for approximately 30 minutes immediately following bilateral contrast injections to the feet. The imaged volume generally included the region from mid-foot to just below the knee. A subset of participants underwent T1-weighted Dixon imaging from knee to mid-thigh. A Dixon imaging approach was chosen for this anatomical region because more robust fat suppression was required.^10^ These images in the thigh were typically collected 30 to 40 minutes after beginning contrast-enhanced imaging.

**Table III:** Magnetic resonance imaging protocol details

Imaging	Anatomy	Orientation	TR/TE, ms	FA(°)	Voxel size (mm)		NSA	Fat suppression	Sense factor (direction)
					Acquired	Reconstructed			
T2-weighted TSE	Leg	Coronal	2800/565	90	2.0 × 2.0 × 3.0	1.2 × 1.2 × 1.5	2	SPAIR	1.6 (RL); 1.6 (AP)
T1-weighted SPGR	Leg	Coronal	3.7/1.6	12	1.0 × 1.0 × 1.0	0.6 × 0.6 × 0.8	1	SPAIR	1.6 (RL); 1.6 (AP)
T1-weighted Dixon	Thigh	Coronal	4.4/1.4, 2.6	10	1.0 × 1.0 × 1.0	0.7 × 0.7 × 0.8	1	–	1.6 (RL); 1.6 (AP)

*AP,* Anteroposterior; *FA,* flip angle; *RL,* right-left directiont; *NSA,* number of signal averaged; *SPAIR,* spectral attenuated inversion recovery; *SPGR,* spoilt gradient echo; *TE,* echo time; *TR,* repetition time; *TSE,* turbo spin echo.

### Data collection, outcome measures, and analysis

#### Anatomy

**Fig 2:**
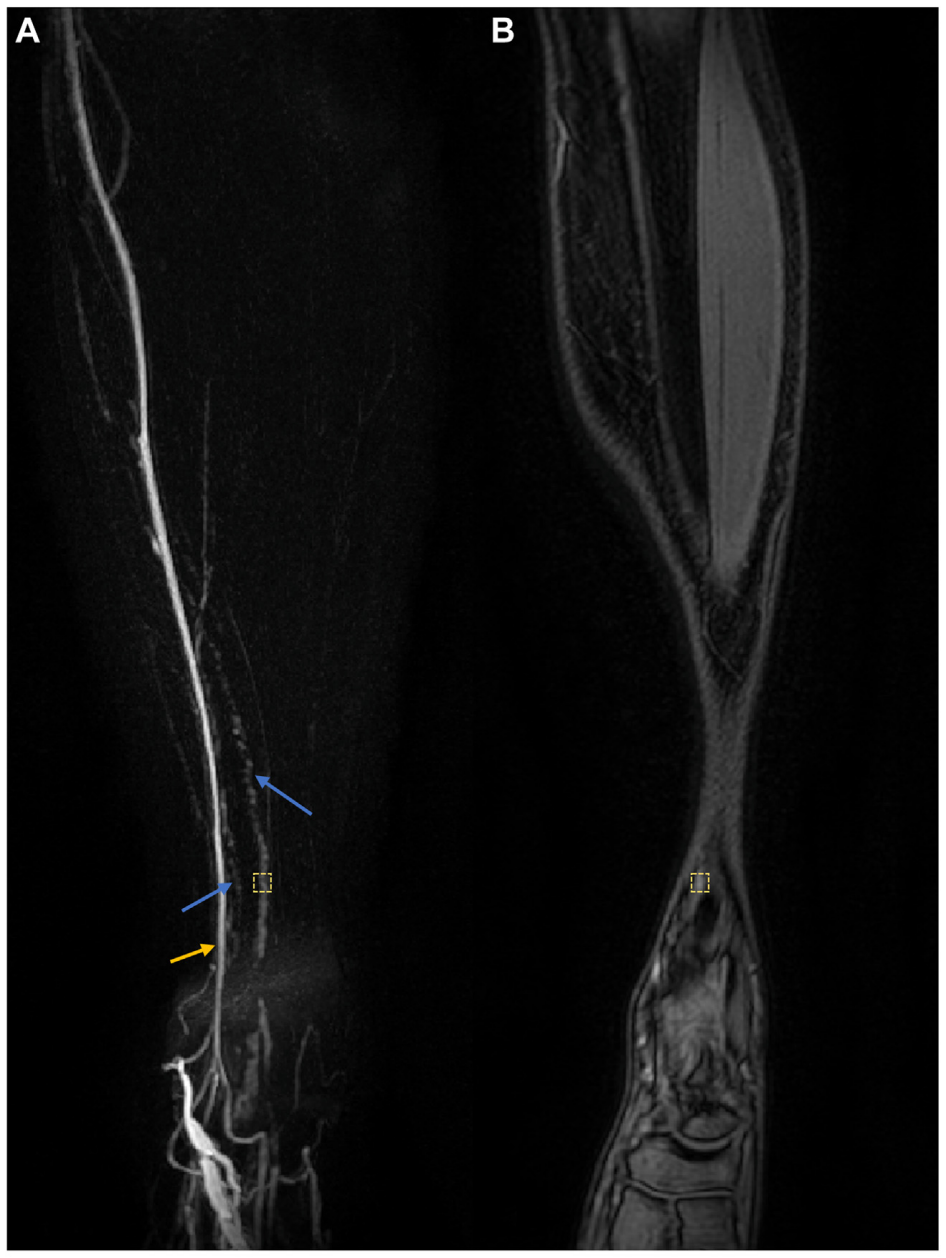
Lymphatic vessel anatomy and function can be visualized. Left leg of a 25-year-old woman showing lymphatic (*blue arrows*) and venous (*orange arrow*) vessels with T1-weighted spoilt gradient echo imaging approximately 20 minutes after contrast administration. **A,** A maximum intensity projection (MIP) is shown after subtraction of the first post-contrast image. **B,** A single coronal image slice of the same timepoint with the approximate location at which lymphatic signal was measured (shown with an *orange hashed box*; enlarged for better visibility).

Lymphatic vessels were identified predominantly by their characteristic beaded and tortuous appearance and were distinguished from veins, which were anticipated to be straighter and of uniform and larger caliber (Fig 2).^11^, ^12^, ^13^, ^14^ Based on the path taken in the limb, lymphatic vessels were classified as belonging to one of four groups, as described by Shinaoka et al^15^: anteromedial, posteromedial, anterolateral, or posterolateral. The identification of vessels was first attempted using the heavily T2-weighted images, before moving on to the dynamic T1-datasets. For the latter, maximum intensity projection (MIP) images were produced in which the first post-contrast image was subtracted from all subsequent dynamic images, as described previously.^16^ The presence of any lymph nodes in the popliteal fossa (popliteal nodes) was also recorded when this region was imaged.

The presence of features such as dermal rerouting, markedly tortuous or transverse lymphatic vessels as seen on T1-weighted images, and hyperintense, fluid-rich, regions of T2-weighted datasets, were considered abnormal findings and recorded. Estimates of the number of lymphatic vessels crossing the anterior ankle and their diameter (taken as the full width at half maximum extent) were also recorded. Lymphatic vessel tortuosity for a single vessel in each leg was estimated after the lymphatic vessel was manually segmented and its centerline extracted using the Vascular Modelling Tool Kit module implemented in 3D Slicer^17^, ^18^, ^19^ as follows:

$$Tortuosity\;Index=\frac{Vessel\;length}{Distance\;between\;vessel\;segment\;endpoints}$$

The chosen vessel had to have been characterized as belonging to the anteromedial pathway and have a minimum length of 200 mm.

#### Lymphatic transport

The rapidity of GBCA drainage, a proxy for lymphatic function, was interrogated in each leg of a subset of five individuals (three male and two female controls; average age, 34.1 ± 9.9 years). The image signal was measured in 3 × 3 × 3 voxel regions centered over a lymphatic vessel just superior to the ankle (Fig 2) and the time from contrast injection to the peak signal (*T*_*peak*_) estimated. The lymphatic signal was normalized to the adjacent muscle to account for signal drift across the time series. The same procedure was also performed in the great saphenous vein just above the ankle and compared with the lymphatic vessels via an independent samples *t* test. All statistical analyses were conducted with SPSS Statistics, version 29.0 (IBM Corp), and *P* < .05 was considered the threshold for statistical significance.

## Results

MRL was successful in all participants without complications. The slight discomfort of injections was well tolerated due to the use of a local anesthetic.

### Injection dose optimization

Inspection of the images from the 18 limbs studied (2 injected with 0.02 mL/mL of GBCA-containing solutions, 6 with 0.1 mL/mL, and 10 with 0.45 mL/mL) demonstrated lymphatic structures in all those administered the 0.45 mL/mL GBCA formulation compared with only one half at 0.1 mL/mL and none at 0.02 mL/mL. In the limbs receiving the highest GBCA concentration, both veins and lymphatic vessels appear brighter (Fig 1). The 0.45-mL/mL protocol was adopted as the dose regimen for the subsequent participants. All subsequent results refer to those limbs administered with this GBCA concentration and exclude those nine individuals imaged as part of the contrast optimization process.

### Main imaging study

#### Leg

After contrast optimization, 16 additional individuals were recruited (7 men and 9 women), for 32 leg examinations. A single limb was excluded leaving 31 for analysis. This exclusion was due to a technical fault, presumed to originate from poor contact between the coil connector and the scanner, which led to severe signal loss affecting a single limb. In all but a single limb, the lymphatic vessels could be visualized during the dynamic T1-weighted imaging. In none of the 31 healthy limb datasets could lymphatic vessels be observed on T2-weighted imaging.

**Fig 3:**
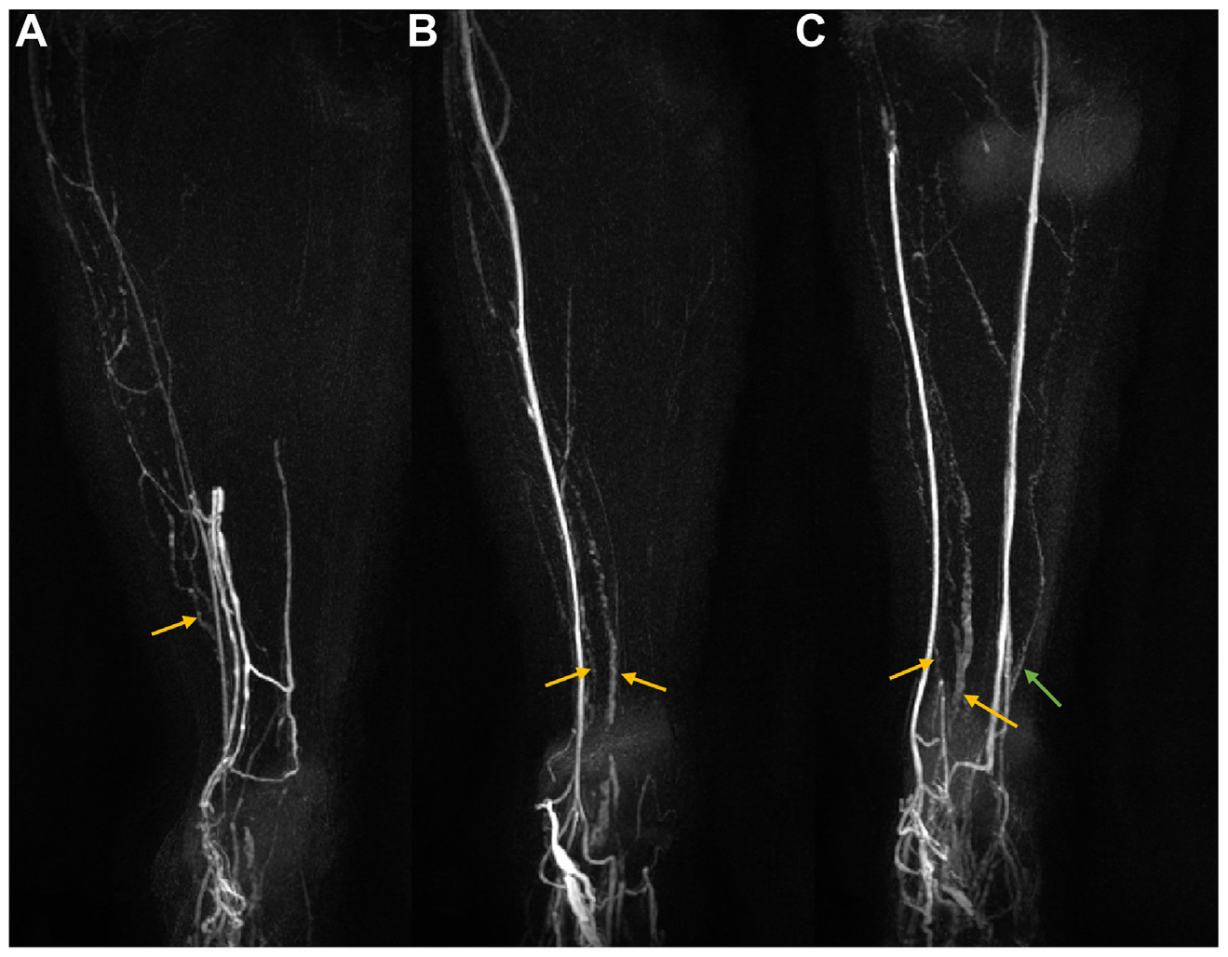
Example images demonstrating a range of vessel numbers and both anteromedial and anterolateral pathways. Coronal subtraction maximum intensity projection (MIP) images for the left leg of three female participants showing one (**A**), two (**B**), and three (**C**) lymphatic vessels observed at the level of the ankle approximately 20 minutes after T1-weighted post-contrast imaging began. **C,** This leg also demonstrates clear anterolateral lymphatic drainage (*green arrow*). *Orange arrows* indicate anteromedial vessels. All limbs also show clear venous enhancement.

The number of vessels noted crossing the anterior ankle ranged from 0 to 4 (mean ± standard deviation, 2.16 ± 0.93; modal count, 3; Fig 3, *A-C*). The predominant drainage pathway observed was the anteromedial (29 of 31 legs), with the anterolateral the only other pathway observed (6 of 31 legs; Fig 3, *C*). The number of enhancing lymphatic vessels and pathways for each participant are listed in the Supplementary Table (online only). The mean vessel diameters were measured at 2.47 ± 0.50 mm (range, 1.56-3.75 mm), and all detected lymphatic vessels resided superficially in the leg (<2 cm from the closest skin boundary).

**Fig 4:**
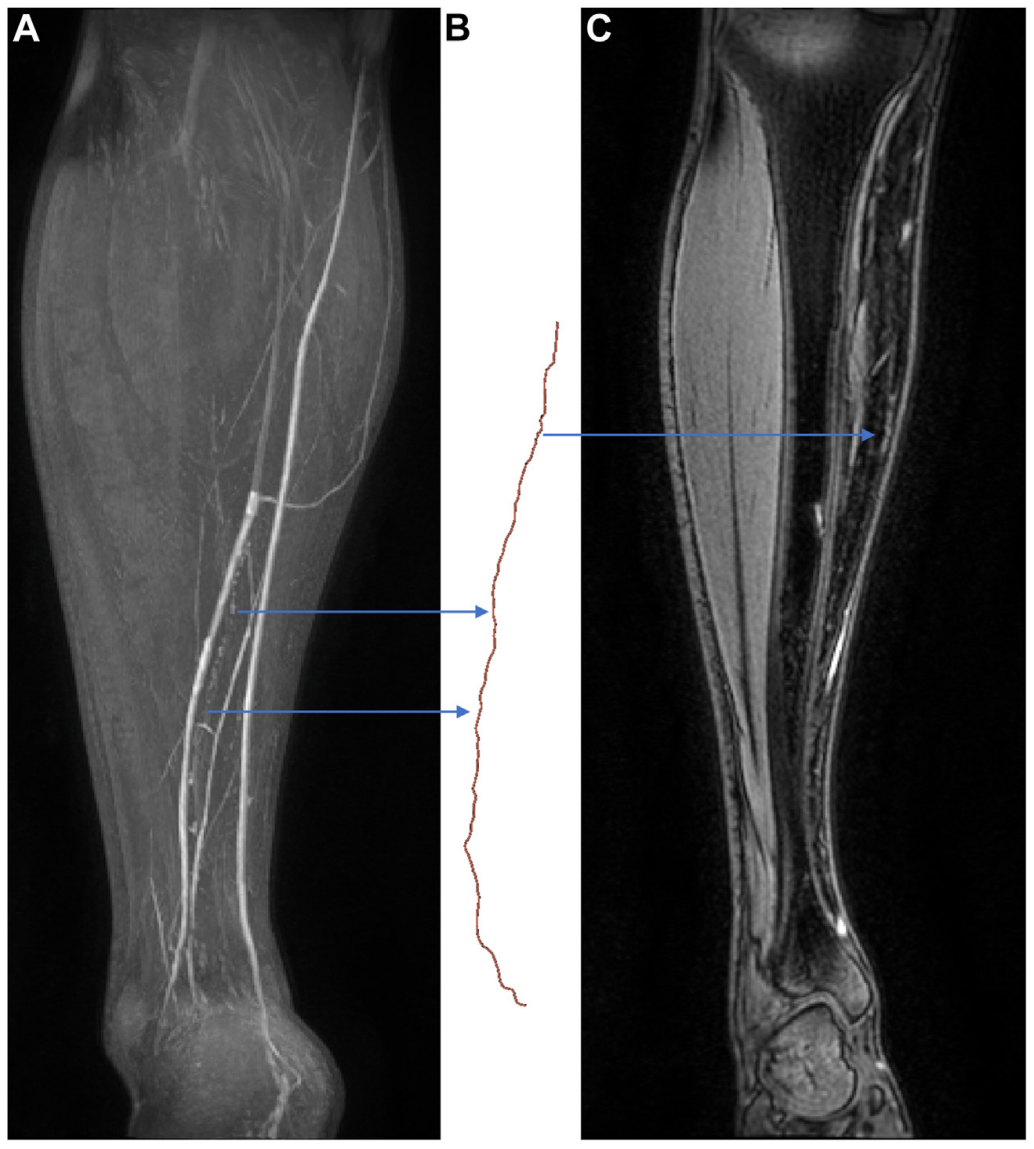
Vessel segment analyzed for tortuosity. Post-contrast images of the left leg of a 31-year-old man. Maximum intensity projection (MIP; **A**) and single slices (**C**) demonstrating the lymphatic vessel, which was segmented (**B**), and whose tortuosity was measured at 1.04. Note how the lymphatic becomes obscured by overlying blood vessels in the MIP (**A**) but can still be clearly observed in the individual coronal slice (**C**). Supplementary Videos 1 and 2 display vessel segment and subtraction MIP images for this limb with rotational projections.

No transverse lymphatic vessels, regions of dermal rerouting, or any potential signatures of lymphatic dysfunction were noted, and no lymphatic vessels appeared abnormally tortuous. The mean tortuosity ± standard deviation was measured at 1.09 ± 0.03 in 27 anteromedial vessels, with vessels in two limbs excluded from analysis due to lengths <200 mm (Fig 4; Supplementary Videos 1 and 2 demonstrate 360° coronal projections of the same vessel segment).

#### Thigh

Of those participants for whom imaging above the knee was performed (26 limbs), in just 6 limbs could the lymphatic vessels be confidently detected using the post-contrast T1-weighted images (data not shown). In all cases, these vessels were observed progressing superficially up the medial knee and thigh toward the inguinal region. No popliteal or any other lymph nodes were observed in any of the participants.

### Fluid accumulation

**Fig 5:**
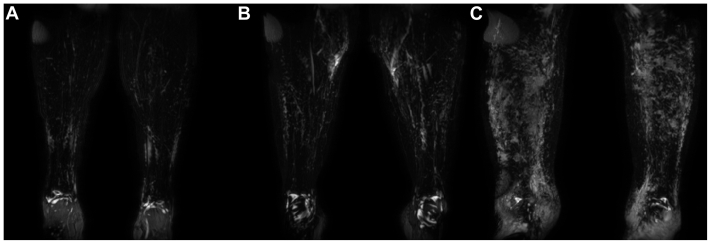
Examples demonstrating a range of T2-weighted images from controls and lymphedema patients. Single slices of heavily T2-weighted images of the mid-leg for 31- (**A**), 37- (**B**), and 27- (**C**) year-old women with identical image windowing and obtained using the same imaging protocol. The 37-year-old healthy control participant showed the most extensive hypointense regions in T2-weighted images of any control included in this study (**B**). **C,** A T2-weighted scan from a similarly aged lymphedema patient (diagnosed with WILD [warts, immunodeficiency, and lymphatic dysplasia] syndrome^20^), with extensive high-signal, fluid-rich, regions throughout the entire limb. **B,** Based purely on a physical inspection of the limbs, the clinician performing the contrast injection suggested that an undiagnosed lipedema could be present, which might explain the increased fluid signal.

A single individual did not undergo this portion of imaging due to timing constraints. In the remaining 29 available limb datasets, most participants did not demonstrate substantial areas of a high signal, indicative of static fluid, beyond that noted within the joints. A single individual's limbs were noteworthy for having demonstrated hyperintense regions throughout the limb, with bilateral regions of particularly high signal in the medial mid-leg (Fig 5).

### Lymphatic transport

**Fig 6:**
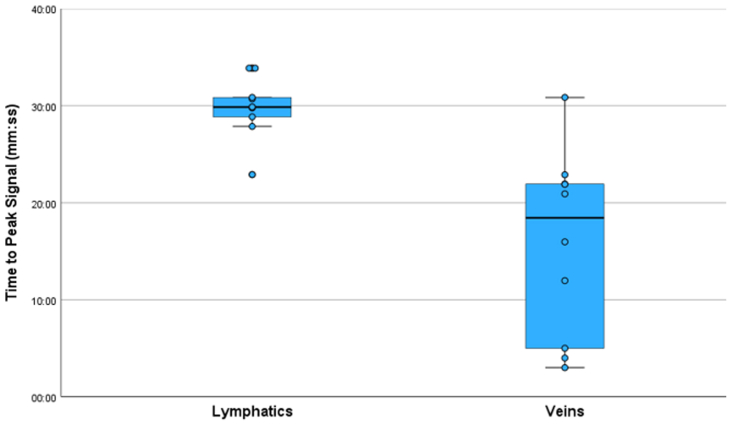
Time to peak signal in lymphatic and venous vessels. Boxplot displaying the time elapsed between the start of contrast injection in a limb and the time at which the peak signal was measured in either an anteromedial lymphatic vessel (eg, the region shown in Fig 2) or the great saphenous vein just superior to the ankle. In the 10 vessels assessed, the lymphatic signal peaked significantly later on average than did the venous signal (mean ± standard deviation, 29 minutes, 50 seconds ± 9 minutes, 29 seconds vs 15 minutes, 50 seconds ± 3 minutes, 6 seconds). Note that 4 of 10 of the lymphatic vessels the peak signal was observed in the final imaging phase.

A significantly quicker peaking of the signal was observed in the veins than in the lymphatic vessels (mean ± standard deviation *T*_*peak*_, 15 minutes, 50 seconds ± 3 minutes, 6 seconds vs 29 minutes, 50 seconds ± 9 minutes, 29 minutes, respectively; *P* < .05; Fig 6). Additionally, in 4 of 10 control limbs, the lymphatic signal peaked in the final imaging phase compared with only a single incidence of this recorded in the veins. The peak venous signal, normalized to the muscle, was also higher compared with that for the lymphatic vessels (mean ± standard deviation, 1.41 ± 0.46 and 2.71 ± 0.62 in the lymphatic vessels and veins, respectively; *P* < .05). Average normalized uptake curves for veins and lymphatic vessels are displayed in the Supplementary Fig (online only).

## Discussion

### Summary

Various lymphatic imaging techniques, including ICG-L and LS, have been successfully deployed to image the lymphatic vessels after injections of an external agent into the feet. These techniques, however, are limited to producing two-dimensional images of the lymphatic vessels, with poor spatial resolution in the case of LS and poor penetrance for ICG-L. MRL appears well placed to overcome these shortcomings; however, visualizing healthy lymphatic vessels with MRL has been reported as problematic.^6^ In this study, we investigated whether MRL is capable of depicting and estimating markers of function within healthy leg lymphatic vessels at 3.0 T. We aimed to provide information related to normal lymphatic anatomy and physiology and demonstrate features that might be beneficial in the detection of lymphatic abnormalities and arriving at a lymphedema diagnosis. Enhancement of lymphatic vessels belonging to the anteromedial or anterolateral drainage routes was observed in all but a single limb after interdigital injections of a GBCA in the forefoot, confirming that MRL is a robust tool for depicting lymphatic vessels in healthy legs. Furthermore, measurements related to lymphatic shape and ability to transport the administered GBCA were demonstrated within the leg. Depiction of lymphatic vessels in the thigh was not commonly observed (6 of 26 thighs), a result we believe is in part due to the long delay (>30 minutes) between contrast injection and imaging this region.

### Contrast optimization

The depiction of normal lymphatic anatomy and estimates of their function are key in improving the utility of MRL for detecting abnormalities; however, failure to visualize lymphatic vessels in healthy subjects has been reported in multiple studies.^21^, ^22^, ^23^ In addition, contrast enhancement of veins can lead to ambiguity in the identification of lymphatic vessels, and concerns exist about the safety of GBCA. We, therefore, explored the effect of the GBCA dose on the visibility of lymphatic vessels in a subset of our participants. Two attempts administering 0.02 mL/mL, which had previously been administered in the arms of individuals with breast cancer-related lymphedema and controls to successfully reduce the effect of venous enhancement,^24^ elicited no clear lymphatic enhancement in our study, and no further attempts with this protocol were attempted. At a concentration of 0.1 mL/mL, the venous signal was reduced compared with 0.45 mL/mL. However, there was also a major loss of lymphatic signal that was considered too costly to warrant adoption of this reduced GBCA protocol. We did not try to administer GBCA undiluted (ie, administered with a local anesthetic but without further dilution) given that in all limbs administered with 0.45 mL/mL lymphatic vessels could be observed. Thus, increasing the total GBCA volume administered was deemed unnecessary. However, this does prevent us from commenting on whether at 0.45 mL/mL the venous signal is reduced relative to the lymphatic vessels compared with when GBCA is given undiluted. Studies exploring optimal contrast injection protocols (eg, contrast agent formulation, dose, local massage), balancing the tradeoff between safety and the lymphatic and venous signal are still required. Also, although the risk of an adverse event is low, the total GBCA dose administered should always be as low as is practical.

### Normal vs abnormal lymphatic vessels

**Fig 7:**
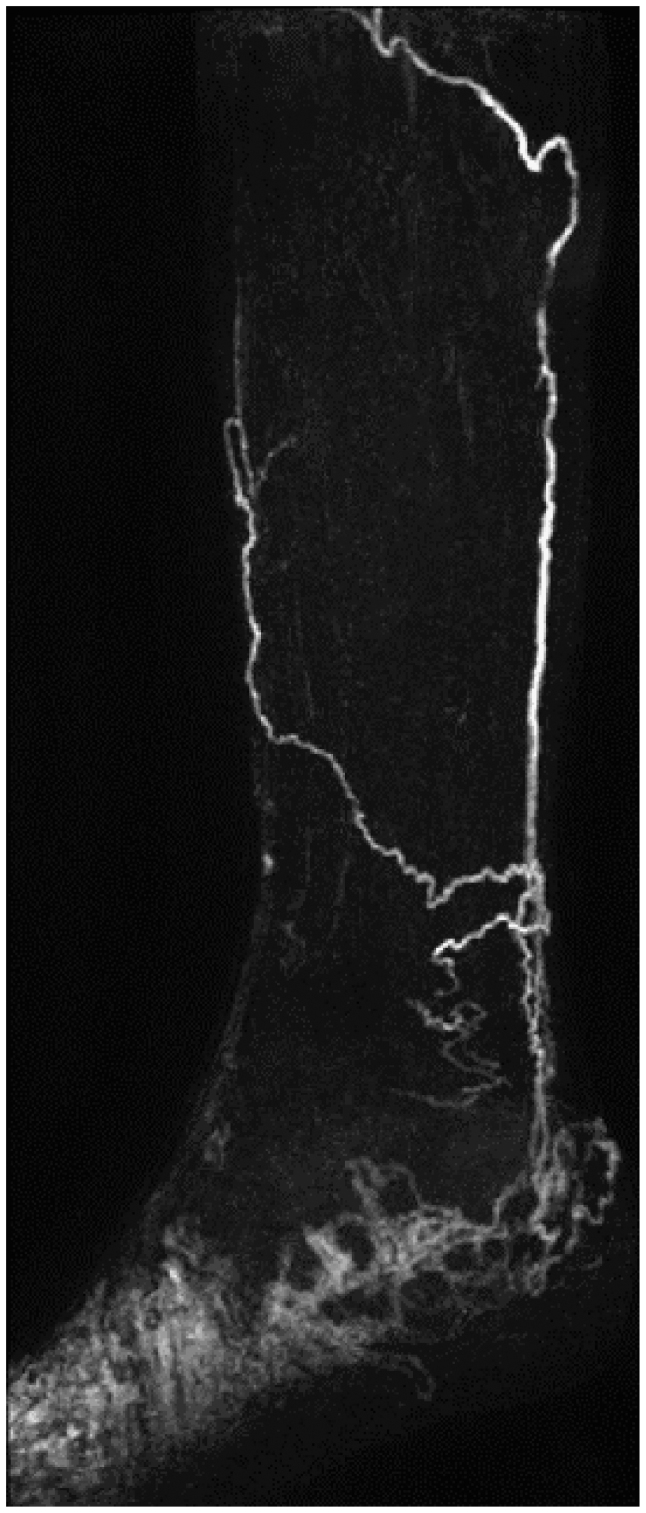
Abnormal tortuous lymphatic vessels in a lymphedema patient. Sagittal T1-weighted subtraction maximum intensity projection (MIP) for a 48-year-old man with a genetically confirmed Milroy diagnosis, demonstrating abnormal-appearing lymphatic vessels and lymphatic vessels traversing the posterior of the limb. Image obtained using interdigital contrast injection and moderately T1-weighted three-dimensional (3D) spoilt gradient echo imaging, as described in this study.

Based on the work by Shinaoka et al*,*^15^ who imaged nonlymphedematous cadavers’ lower limbs with ICG-L and CT, we anticipated forefoot injections to elicit contrast drainage via vessels belonging predominantly to the anteromedial pathway. We confirmed this, with enhancing anteromedial lymphatic vessels seen in most limbs (29 of 31 limbs). In approximately one fifth of cases, including a single limb not demonstrating anteromedial lymphatic enhancement, anterolateral vessels were observed. The presence of anterolateral lymphatic enhancement should therefore not necessarily be considered pathological collateralization after forefoot contrast injections. Again, in agreement with the cadaver study,^15^ we observed that all enhancing vessels remained superficial and, hence, anticipated the finding that no enhancement of popliteal lymphatic nodes would be observed. However, we, and others, have noted collateral posterior lymphatic drainage after interdigital contrast administration in cases of lymphedema (Fig 7), with Soga et al^25^ also reporting popliteal node enhancement when imaging lymphedema patients’ lower limbs at 1.5 T.

The results of our investigation of lymphatic tortuosity confirm that, although the collector vessels of lymphedema patients are often reported as being abnormally tortuous, the lymphatic vessels of healthy individuals appear relatively linear by comparison.^26^, ^27^, ^28^ We have built on the descriptive results of prior studies by computing the absolute tortuosity in lymphatic vessel segments, a value that is simple to compute and could be used to better stratify normal and abnormal lymphatic vessels. Further highlighting the readily quantifiable nature of MRL, we recorded the elapsed time between the beginning of the GBCA injection and the signal peak in regions of anteromedial lymphatic vessels and the adjacent great saphenous vein. The signal in the lymphatic vessels peaked significantly later than in the veins, as has been previously reported,^24^ and showed lower variability in *T*_*peak*_. This clustering might be somewhat artificial given that in 4 of 10 vessels the signal was still increasing at the cessation of imaging. This will also likely have reduced the true difference in *T*_*peak*_ between vessel types, further highlighting the potential for this measurement to be used to separate lymphatic and venous structures. Additionally, this assessment has been used to compare lymphatic contrast uptake rates in cases of lymphedema distichiasis syndrome compared with healthy controls, in whom the lymphatic signal peaked significantly more rapidly in the patients with lymphedema distichiasis syndrome.^29^

The healthy nature of our cohort also meant that in all cases, T2-weighted imaging highlighted no lymphatic vessels and few areas of fluid accumulation outside the joints, both of which have been reported in studies imaging lymphedema patients.^30^^,^^31^ A single individual demonstrated particularly extensive intense regions on T2-weighted imaging compared with the other controls but substantially less than can be observed in many lymphedema patients (Fig 4). It was remarked by the clinician performing the GBCA injection (P.S.M.) during the preimaging examination that he believed this individual showed clinical signs indicative of lipedema, which might explain the difference in image appearance for this case. This individual demonstrated normal-appearing lymphatic vessels, however, and removing their data from the analysis had very little effect, with an average vessel count at the ankle of 2.21 ± 0.94 and diameter of 2.48 ± 0.50 mm (vs 2.16 ± 0.93 and 2.47 ± 0.50 mm, respectively, before exclusion).

### Strengths and weaknesses of MRL

As stated, MRL has some key advantages over alternative imaging techniques, including improved spatial resolution and the nonionizing nature compared with LS and an ability to visualize lymphatic vessels beyond the most superficial compared with ICG-L. In addition, MRL is able to depict both the lymphatic vessels and the soft tissues three-dimensionally. There exist some weaknesses with the technique that need addressing. Foremost among these is that intradermal GBCA injection causes both lymphatic and venous enhancement, which can lead to difficulty in assigning enhancing vessels as being of lymphatic or venous origin. The generally smaller and discontinuous appearance of the lymphatic vessels aids in their identification, and we also found the use of subtraction images further assists with differentiation.^16^ The use of prospective measures to combat venous enhancement might be of particular benefit in lymphedema patients in whom dilated lymphatic vessels have been observed.^12^ Administration of an ultra-small superparamagnetic iron oxide agent either into the bloodstream, reducing the blood's T2 value and signal, or as an interstitial agent that is preferentially trafficked into the lymphatic vessels shows great potential.^32^^,^^33^ Despite these promising results, we have yet to adopt this agent for use in our own studies because, at the time of writing, the agent remains unlicensed for use as an MR contrast agent in the United Kingdom. Our observation of a more rapid wash-in and wash-out of the contrast agent in the great saphenous vein compared with the proximal lymphatic vessels might offer an additional avenue for improving identification of venous and lymphatic structures based on their uptake characteristics. Whether this difference holds for lymphedema patients is yet to be established. This difference also helps to explain the benefit of subtracting the initial post-contrast image from all subsequent images regarding lymphatic visibility: any venous enhancement beyond this initial imaging phase is likely to be small compared with the change in lymphatic signal over time.

At present, MRL has only been used to demonstrate the larger lymphatic vessels with T1-weighted imaging resolutions typically of the order of 1 mm isotropic.^9^ To image smaller vessels and the precollector vessels proximal to contrast injection sites, further optimization of the imaging and contrast injection protocol might be required. It has been noted, for example, that with higher concentrations of GBCA injection, a loss of signal at the injection site can occur.^24^ Imaging at higher field strengths might, therefore, be required for these vessels, given the resultant boost to the image signal.

MRL is also limited by all the contraindications that prevent participants from undergoing any magnetic resonance-based examination (eg, implanted medical device, claustrophobia). Those with a history of allergies to contrast agents or other medications might also be prevented from proceeding with a contrast-enhanced study. However, many studies have demonstrated the utility of noncontrast imaging in assessing lymphatic disease.^8^^,^^28^^,^^34^ In any case, the administration of GBCA intradermally is considered off-label use; thus, care must be taken by the administering clinician to thoroughly consider the risks and benefits of GBCA injections. However, as with many previous studies, we report no adverse events associated with this type of contrast injection.^13^^,^^35^

### Study weaknesses and future work

We acknowledge that this study is limited by several factors. We recruited healthy controls from a local population that might not be indicative of the wider population and did not image children. Second, we performed quantitative assessments of the lymphatic uptake rates and vessel tortuosity on only a single vessel in the limbs studied. Future studies would benefit both from exploring all viable vessels and from exploring the repeatability of these measurements. The choice of metrics also might not be the most sensitive for detecting pathological changes. Curvature-based estimates might prove a better probe of changes in lymphatic vessel morphology than tortuosity, as was recently shown in a study exploring blood vessel changes associated with coronary artery disease.^36^ This study is also limited by the lack of patient data. We are currently in the process of collecting patient datasets, for which this work relating to healthy controls will be of great value as a comparison group without signs of lymphatic disease.

Lymphatic dysfunction has been associated with fat deposition, in addition to increased fluid, and MRI has been used to show this within individuals with a breast cancer-related lymphedema diagnosis and for whom the nonedematous limb was used as an internal comparison to the affected side.^37^ In the same report, reduced T2 values were seen to correlate with increased fat deposition, providing further evidence of a disrupted tissue environment in cases of lymphedema and how MRI can be used to assess this. Although we did not attempt to quantify the fat volume or fraction in this study, fat-sensitive Dixon imaging, both within the leg and thigh, is now performed for our unaffected and lymphedematous research subjects, and we will explore this tissue compartment in future studies.

We did not explore the effect of alternative injection sites in relation to the depiction of deep lymphatic pathways. The ability to demonstrate these deeper lymphatic vessels is considered a particular differential between MRL and ICG-L. The adoption of contrast injection to the lateral malleolus, as is performed in some ICG-L studies,^38^ should be explored in the future to determine the additional benefits of imaging the lymphatic vessels with MRL following these injections. This should be performed first in healthy individuals, given that posterior lymphatic enhancement from the forefoot injections has been observed in some lymphedema patients with MRL.^25^ Additionally, the effect of imaging the thigh sooner after contrast injection should be explored to determine whether this improves the visibility of lymphatic vessels in the region or if higher GBCA concentrations or injected volumes are required.

## Conclusions

Before MRL becomes a clinical and research mainstay for investigating lymphatic dysfunction it must be shown to be well tolerated by, and to provide robust demonstration of, the lymphatic anatomy within healthy subjects. This study illustrates that with an appropriate contrast injection and imaging protocol, lymphatic vessels can be routinely identified in the limbs of healthy controls and simple quantitative values associated with lymphatic anatomy and drainage can be obtained. These findings were established in a broad age range of both male and female controls, which we believe to be of particular significance given that the manifestation of lymphedema (primary or secondary) affects both men and women at any age.

Confirming the appearance and behavior of healthy lymphatic vessels on MRL is a valuable step toward establishing a comparison group for cases of lymphatic dysfunction. Moreover, it shows that the examination is well tolerated and, hence, amenable to deployment in both the diagnosis of lymphatic disease and tracking the course of disease. The quantifiable nature of magnetic resonance imaging (eg, estimation of tissue compartment volumes, vessel sizes, and rate of contrast uptake) also position it well as an appropriate method for assessing treatment response. However, further research of both healthy controls and patients is needed to identify suitable biomarkers and to develop physiological models of GBCA movement, as has been achieved with contrast-enhanced magnetic resonance imaging in oncology.

## Author contributions

Conception and design: MM, KG, PM, PO, FH

Analysis and interpretation: MM

Data collection: MM, GB, BH, JP, PM

Writing the article: MM

Critical revision of the article: GB, BH, JP, KG, PM, PO, FH

Final approval of the article: MM, GB, BH, JP, KG, PM, PO, FH

Statistical analysis: Not applicable

Obtained funding: KG, PM, PO, FH

Overall responsibility: MM

## Disclosures

None.

## Figures and Tables

**Supplementary Fig (online only):**
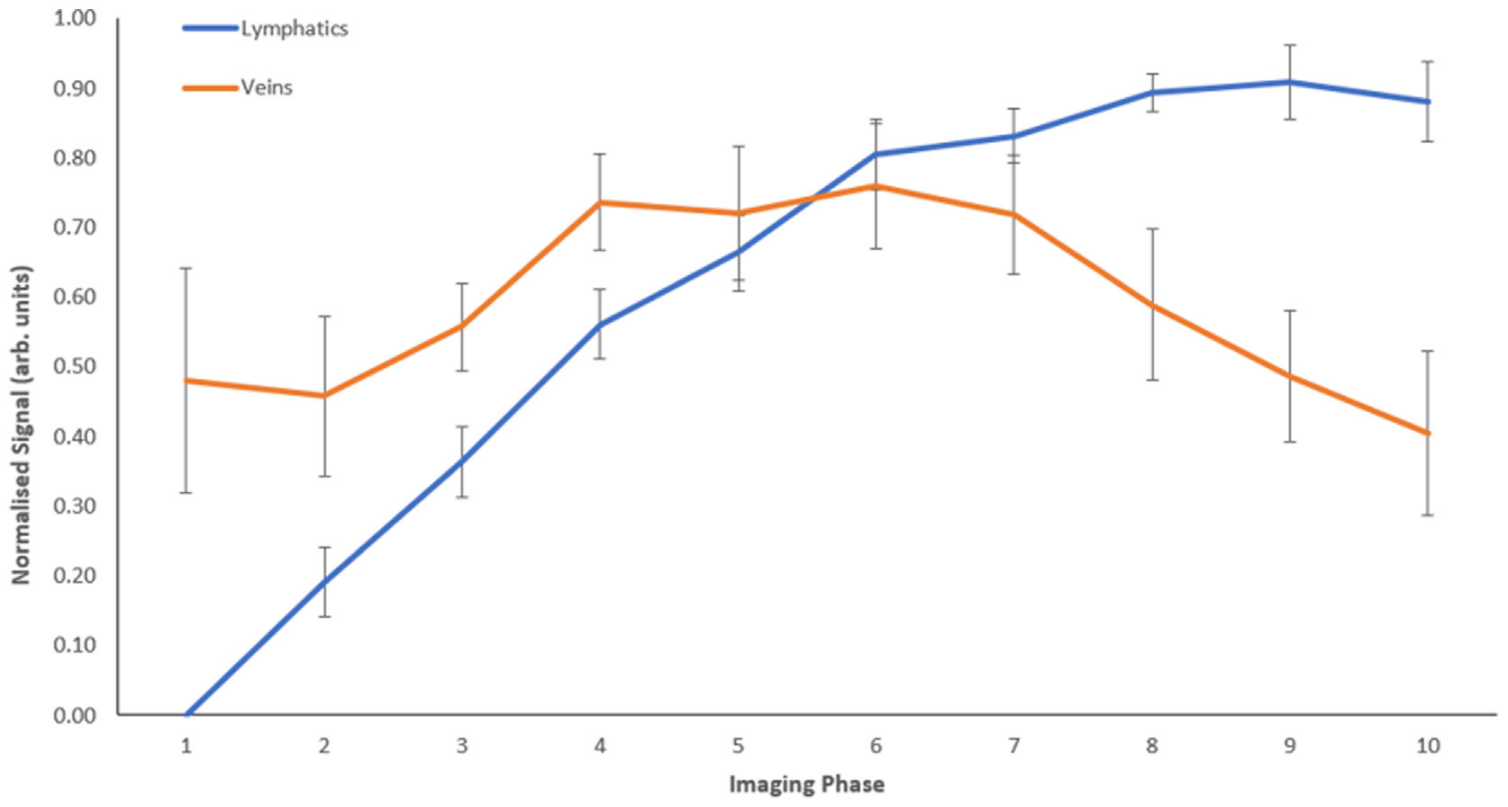
Normalized average uptake curves in lymphatic system and long saphenous vein showing the gradual increase in lymphatic signal across the 10 post-contrast imaging phases (approximately 30 minutes) compared with a more rapid wash-in and wash-out seen in the veins. For each vessel, the signal was transformed to take a value between 0 and 1 before averaging across the 10 vessels. Error bars indicate standard error of the mean.

**Supplementary Table (online only):** Lymphatic vessel pathways and number observed crossing the anterior ankle for the 16 participants imaged after contrast optimization

		Vessels crossing ankle, No.		Pathways Present			
				Anteromedial		Anterolateral	
Participant No.	Age, years	Left	Right	Left	Right	Left	Right
1	29.1	3	3	X	X	–	X
2	69.0	3	1	–	X	X	–
3	41.1	2	3	X	X	–	X
4	25.9	1	1	X	X	–	–
5^a^	53.4	3	NA	X	NA	–	NA
6	31.7	3	1	X	X	–	–
7	37.3	1	2	X	X	–	–
8	47.0	1	2	X	X	–	–
9	36.5	2	2	X	X	–	–
10	37.5	1	3	X	X	–	–
11	55.4	3	2	X	X	–	–
12	42.1	0	2		X	–	–
13	40.7	3	3	X	X	X	–
14	33.2	2	4	X	X	–	X
15	25.8	2	2	X	X	–	–
16	51.2	3	3	X	X	–	X

*NA,* Not applicable.

a During imaging, the right leg of participant 5 suffered severe signal loss and could not be analyzed.

## Appendix (online only)

Supplementary Video 1 Post-contrast rotational maximum intensity projections of the left leg of a 31-year-old man demonstrating anteromedial lymphatic vessels (see also Fig 4).

Supplementary Video 2 Video demonstrating a segmented anteromedial lymphatic vessel of the left leg of a 31-year-old man (also shown in Fig 4 and Supplementary Video 1). Lymphatic vessels were segmented in 10 limbs to estimate tortuosity (the ratio of their length to the distance between their end points). The segmentations, and this video, were created using the open-source 3D Slicer software, version 5.2.2.
